# 4-Cyanamidobenzenesulfonamide derivatives: a novel class of human and bacterial carbonic anhydrase inhibitors

**DOI:** 10.1080/14756366.2022.2138367

**Published:** 2022-10-28

**Authors:** Morteza Abdoli, Alessandro Bonardi, Claudiu T. Supuran, Raivis Žalubovskis

**Affiliations:** a Faculty of Materials Science and Applied Chemistry, Institute of Technology of Organic Chemistry, Riga Technical University, Riga, Latvia; b Neurofarba Department, Università degli Studi di Firenze, Florence, Italy; c Latvian Institute of Organic Synthesis, Riga, Latvia

**Keywords:** Carbonic anhydrase, cyanamides, sulphonamides, Mammaliicoccus (*Staphylococcus*) *sciuri*, *Salmonella enterica* (serovar Typhimurium)

## Abstract

A one-pot two-step protocol was developed for the synthesis of a series of novel 4-cyanamidobenzenesulfonamides from easily accessible methyl (4-sulfamoylphenyl)-carbamimidothioate. The new sulphonamides were investigated as inhibitors of the enzyme carbonic anhydrase (CA, EC 4.2.1.1), the human (h) cytosolic isoforms hCA I, II, VII, and XIII, as well as three bacterial enzymes belonging to the β-CA class, MscCA from *Mammaliicoccus (Staphylococcus) sciuri* and StCA1 and StCA2, from *Salmonella enterica* (serovar *Typhimurium*). The human isoforms were generally effectively inhibited by these compounds, with a clear structure-activity relationship privileging long aliphatic chains (C6, C7 and C18) as substituents at the cyanamide functionality. The bacterial CAs were also inhibited by these compounds, but not as effective as the hCAs. The most sensitive enzyme to these inhibitors was StCA1 (K_I_s of 50.7 − 91.1 nM) whereas SscCA was inhibited in the micromolar range (K_I_s of 0.86–9.59 µM).

## Graphical abstract

**Figure:**
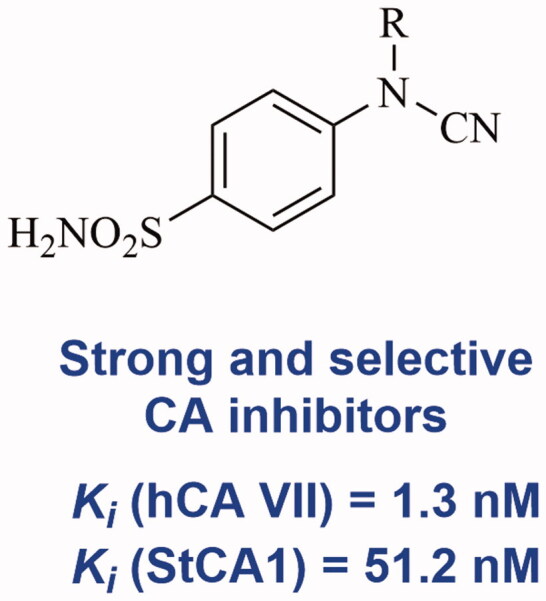

## Introduction

Sulphonamides are one of the crucial classes of bioactive compounds that played a pivotal role in the field of drug discovery and development due to their diversified pharmacological activities, with more than seventy FDA-approved medications containing one or more sulphonamide motifs in their structure^1^. The best known and striking biological features of primary sulphonamide-containing compounds is their ability to inhibit the metalloenzyme carbonic anhydrase (CA, EC 4.2.1.1) by binding to the zinc ion within its catalytic site^2^. CAs are widespread in all types of organisms, in the three domains of life, Archaea, Bacteria and Eukarya^3^. Presently, 15 different CA isoforms (CA I, II, III, IV, VA, VB, VI, VII, VIII, IX, X, XI, XII, XIII, and XIV) were described in humans/primates, 12 of which being able to catalyse the reversible interconversion of CO_2_ to HCO_3_^−^ and a proton, whereas CA VIII, X, and XI are devoid of CO_2_ hydrase activity^3^. This reaction plays fundamental roles in many physiological and pathological events, including respiration and CO_2_ transport, secretion of electrolytes, pH and other ions homeostasis, bone reabsorption, calcification, tumorigenesis, etc.^4^. Therefore, the involvement of various CA isoform in such processes can be and is exploited for the development of different types of therapeutic agents, among which diuretics, anti-glaucoma, antiepileptic, anti-obesity and anti-tumour medications^5–7^.

CAs are on the other hand widespread in bacteria, with at least four CA genetic families being present in these organisms, the α-, β-, γ-, and ι-class enzymes, of the eight CA classes described so far^8^^,^^9^. Many members of such CAs were discovered, isolated and characterised extensively, mainly in pathogenic bacteria, in the search of inhibitors acting as antibiotics with a diverse mechanism of action compared to the clinically used such agents^8–11^. Indeed, recent studies demonstrated that bacterial infections difficult to treat due to the drug resistance problems, such as those provoked by vancomycin-resistant *Enterococci* or *Neisseria* spp., can be managed by using CA inhibitors of the sulphonamide type^9^. Such promising results opened new directions in the design of CAIs selective for the various vertebrate isoforms, but also for compounds that might specifically target the bacterial over the host enzymes^2^^,^^4^^,^^8–11^.

The cyanamide moiety is one of the bioactive motifs which is featured in a large number of biologically active molecules as well as some drugs, possessing a wide range of activities, such as antihistamine^12^, anti-ischemia^13^, anti-tumour^14^, anti-inflammatory^15^, and anti-apoptotic^16^ effects. Without a slight doubt, expanding the database of carbonic anhydrase inhibitors by appending unique functionalities at aromatic ring periphery of arenesulphonamide scaffolds (mostly para position of well-studied benzenesulphonamide) is highly desirable for the development of isoform selective CAIs and has been the subject of large number of studies in recent years. Cyanamide (–NCN) moiety is not only one of the well-known pharmacophores but also a versatile building block in the synthesis of various cyclic and acyclic nitrogen-containing compounds. Therefore, synthesis and evaluation of CA inhibitory activity of hitherto unknown 4-cyanamidobenzenesulphonamide derivatives will not only help to expand the database of carbonic anhydrase inhibitors but also may provide useful intermediates which could subject to further manipulations for the preparation of novel and more complex sulphonamide-based CAIs.

**Scheme 1.**
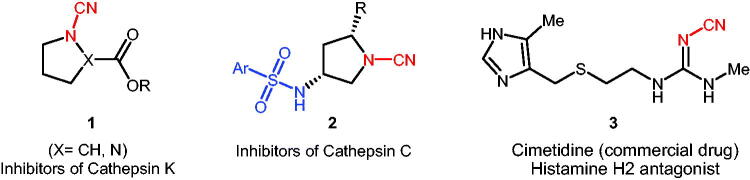
Selected examples of biologically active cyanamide derivatives of types **1–3**.

Additionally, there are several pathological processes which besides carbonic anhydrases other enzymes are also involved in their mechanism. An interesting example is bone resorption. Cathepsin K is considered the major cysteine protease expressed in osteoclasts and has been proposed to play a pivotal role in osteoclast-mediated bone resorption. Like all lysosomal enzymes, cathepsin K also require acidic pH for its optimum activity and it is well documented that CA II facilitates proton production, leading to the acidification of resorption lacunae, trigger cathepsin K activation, and ultimately to bone dissolution. Therefore, inhibition of either CA II or cathepsin K may prevent bone resorption. Similarly, CA VII and cathepsins (S, K) were recently validated as potential therapeutic targets in neuropathic pain, albeit more studies are needed to understand potential relationship between CA VII and cathepsin(s) in this syndrome. On one hand it is well known that (hetero)aryl sulphonamides are the widely used drug class for the inhibition of CAs, on the other hand, cyanamides are one of the main classes of cathepsin inhibitors. Therefore, synthesis of a novel panel of compounds possessing both sulphonamide and cyanamide moieties may help to develop effective multi-target drugs as well as unresisting the mechanism of various pathological processes. Importantly compounds containing electrophilic cyanamide warhead displayed significant inhibitory activities against human cathepsins^17^^,^^18^, inhibiting the lysosomal cysteine protease cathepsin S (CatS)^19^^,^^20^ (Scheme 1).

**Scheme 2.**
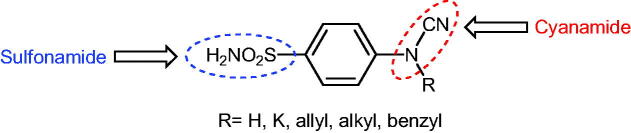
Schematic presentation of compounds studied in this paper.

In this regard, in the next stage, we may study the inhibitory activity of the same set of compounds reported in this paper against cathepsin(s).

In light of the above-mentioned facts, we hypothesised that compounds possessing sulphonamide and cyanamide moieties might show efficient inhibitory activity against various human and bacterial CAs. Thus, in continuation of our interest in the development of selective CA inhibitors (CAIs)^21^, herein, we present the synthesis of novel cyanamide-containing sulphonamides (Scheme 2) and evaluate their capability to inhibit various human and bacterial CAs.

**Scheme 3.**
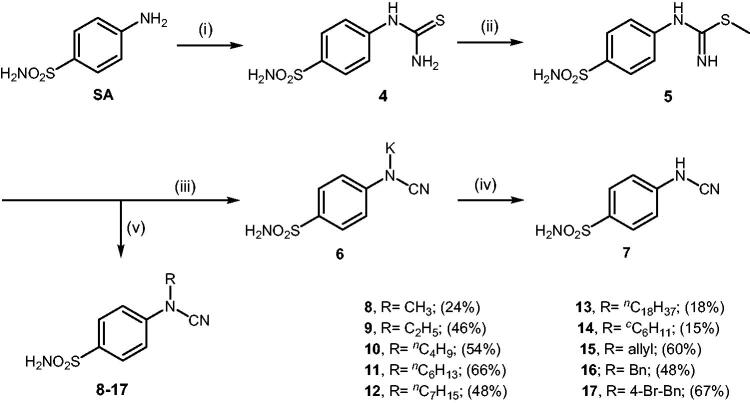
Reagents and conditions: (i) KSCN, aq. 3.5 M HCl, reflux, 3 h, 31%; (ii) MeI, DMF, 40 °C, 2.5 h, 70%; (iii) K_2_CO_3_, DMF, 100 °C, 1.5 h, 89%; (iv) acetic acid (4 equiv.), DMF, RT, 10 min, 59%; (v) (a) K_2_CO_3_, DMF, 100 °C, 1.5 h; (b) RX (X = Br or I), DMF, 40–100 °C, 1–1.5 h.

## Materials and methods

### Chemistry

Reagents, starting materials and solvents were obtained from commercial sources and used as received. Thin-layer chromatography was performed on silica gel, spots were visualised with UV light (254 and 365 nm). NMR spectra were recorded on Bruker 300 spectrometer with chemical shifts values (*δ*) in ppm relative to TMS using the residual DMSO-d_6_ signal (^1^H 2.50; ^13^C 39.52). High-resolution mass spectra (HRMS) were recorded on a mass spectrometer with a Q-TOF micro mass analyser using the ESI technique.

### Synthesis

#### 4-Thioureidobenzenesulfonamide (4)


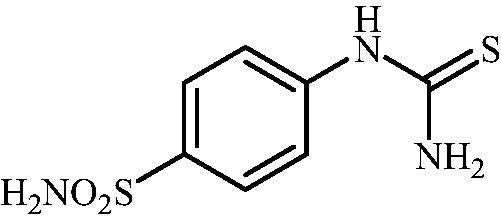


4-Aminobenzensulfonamide (30.00 g, 174.3 mmol) was dissolved in 180 ml of 3.5 M HCl at 70 °C. After cooling to room temperature, KSCN (16.94 g, 174.3 mmol) was added and the mixture was refluxed for 3 h. After cooling to room temperature, the reaction mixture was diluted with ice cooled water. Solids formed were filtered, washed with water, and air dried to afford **9** (12.49 g, 31%) as a white powder.

^1^H NMR (300 MHz, DMSO-d_6_) *δ* = 7.32 (s, 2H), 7.69 (d, 2H, *J =* 8.6 Hz), 7.77 (d, 2H, *J =* 8.6 Hz), 10.02 (s, 1H) ppm ^13^C NMR (75 MHz, DMSO-d_6_) *δ* = 122.8, 127.3, 139.8, 143.9, 182.8 ppm MS (ESI) [M + H]^+^: m/z 232.0.

#### Methyl (4-sulfamoylphenyl)carbamimidothioate (5)


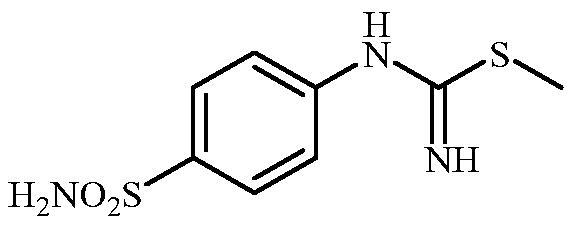


4-Thioureidobenzenesulfonamide (**4**) (300 mg, 1.3 mmol) was dissolved in DMF (4 ml) at room temperature. MeI (0.08 ml, 1.3 mmol) was added to the solution and the mixture was heated at 40 °C for 2.5 h. After cooling to room temperature, it was extracted with EtOAc (3 × 20 ml). The combined organic phases were washed with aq. sat. NaHCO_3_ (2 × 20 ml) and aq. sat. NH_4_Cl in water (1 × 20 ml) and dried over Na_2_SO_4_. Solvent removal *in vacuo* afforded **5** (223 mg, 70%) as a white powder.

^1^H NMR (300 MHz, DMSO-d_6_) *δ* = 2.37 (s, 3H), 6.63 (s, 2H), 6.94 (s, 2H), 7.22 (s, 2H), 7.71 (d, 2H, *J =* 8.4 Hz) ppm ^13^C NMR (75 MHz, DMSO-d_6_) *δ* = 14.2, 122.8, 127.7, 138.0, 153.9, 157.0 ppm HRMS (ESI) [M + H]^+^: *m*/*z* calcd for (C_8_H_12_N_3_O_2_S_2_) 246.0371. Found 246.0372.

#### Potassium cyano(4-sulfamoylphenyl)amide (6)


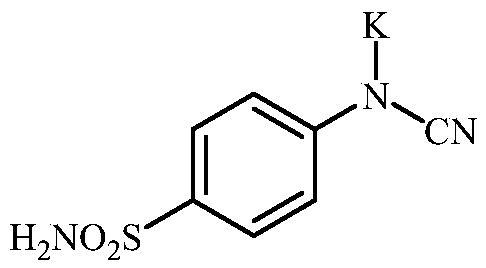


To a solution of methyl (4-sulfamoylphenyl) carbamimidothioate (**5**) (500 mg, 2.04 mmol) in DMF (8 ml) was added K_2_CO_3_ (564 mg, 4.08 mmol,) and the mixture was stirred at 100 °C for 1.5 h. The mixture was cooled to room temperature and precipitated K_2_CO_3_ was removed by filtration. To the solution EtOAc (80 ml) was added and precipitate formed was collected by filtration, washed with EtOAc (20 ml) and air dried to afford **6** (427 mg, 89%) as a white powder.

^1^H NMR (300 MHz, DMSO-d_6_) *δ* = 6.60 (d, 2H, *J =* 8.6 Hz), 6.85 (s, 2H), 7.29 (s, 1H), 7.38 (d, 2H, *J* = 8.6 Hz) ppm ^13^C NMR (75 MHz, DMSO-d_6_) *δ* = 118.0, 125.7, 127.9, 129.0, 160.9 ppm HRMS (ESI) [M – K]^-^: *m*/*z* calcd for (C_7_H_6_N_3_O_2_S) 196.0181. Found 196.0188.

#### 4-Cyanamidobenzenesulfonamide (7)


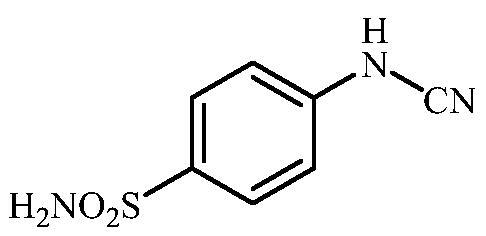


To a solution of methyl (4-sulfamoylphenyl)carbamimidothioate (**5**) (300 mg., 1.22 mmol) in DMF (4 ml) under stirring K_2_CO_3_ (338 mg, 2.44 mmol) was added in one portion. The mixture was stirred at 100 °C for 1.5 h. After cooling to room temperature, the mixture was neutralised by addition of acetic acid (0.28 ml, 4.88 mmol). It was extracted with EtOAc (3 × 20 ml). The combined organic phases were dried over Na_2_SO_4_ and the solvent was removed under reduced pressure. The residue was purified by column chromatography on silica gel (DCM:MeOH, 95:5) to afford **7** (141 mg, 59%) as a white powder.

^1^H NMR (300 MHz, DMSO-d_6_) *δ* = 7.08 (dd, 2H, *J =* 6.7 and 1.9 Hz), 7.28 (s, 2H), 7.79 (dd, 2H, *J* = 6.7 and 1.9 Hz), 10.70 (br s, 1H) ppm ^13^C NMR (75 MHz, DMSO-d_6_) *δ* = 112.3, 115.9, 128.8, 139.0, 142.9 ppm HRMS (ESI) [M + H]^+^: *m*/*z* calcd for (C_7_H_8_N_3_O_2_S) 198.0337. Found 198.0333.

#### 4-(N-Methylcyanamido)benzenesulfonamide (8)


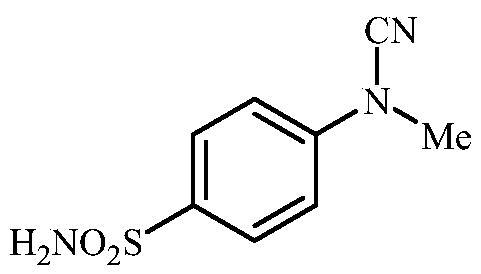


To a solution of methyl (4-sulfamoylphenyl)carbamimidothioate (**5**) (500 mg, 2.04 mmol) in DMF (8 ml) under stirring K_2_CO_3_ (564 mg, 4.08 mmol) was added in one portion The mixture was stirred at 100 °C for 1.5 h. After cooling to room temperature, MeI (0.127 ml, 2.04 mmol) was added and the mixture was stirred at 40 °C for 1 h. The reaction mixture was cooled to room temperature and extracted with EtOAc (3 × 20 ml). The combined organic phases were washed with aq. sat. NaHCO_3_ (2 × 20 ml) and aq. sat.NH_4_Cl (1 × 20 ml) and dried over Na_2_SO_4_. The solvent was removed *in vacuo* and the residue was washed with EtOAc (2 × 10 ml) to afford **8** (103 mg, 24%) as a white powder.

^1^H NMR (300 MHz, DMSO-d_6_) *δ* = 3.42 (s, 3H), 7.32 (d, 2H, *J* = 8.8 Hz), 7.37 (s, 2H), 7.89 (d, 2H, *J =* 8.8 Hz) ppm ^13^C NMR (75 MHz, DMSO-d_6_) *δ* = 37.7, 114.1, 115.8, 128.5, 139.5, 144.2 ppm HRMS (ESI) [M]^+^: *m*/*z* calcd for (C_8_H_9_N_3_O_2_S) 211.0415. Found 211.0419.

#### 4-(N-Ethylcyanamido)benzenesulfonamide (9)


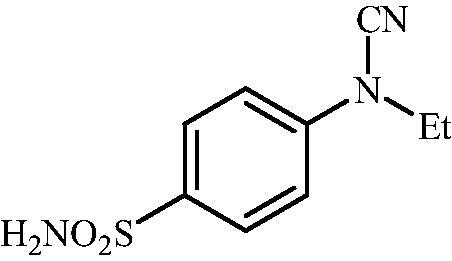


To a solution of methyl (4-sulfamoylphenyl)carbamimidothioate (**5**) (500 mg, 2.04 mmol) in DMF (8 ml) under stirring K_2_CO_3_ (564 mg, 4.08 mmol) was added in one portion The mixture was stirred at 100 °C for 1.5 h After cooling to room temperature, EtI (0.164 ml, 2.04 mmol) was added and the mixture was stirred at 100 °C for 1 h. The reaction mixture was cooled to room temperature and extracted with EtOAc (3 × 20 ml). The combined organic phases were washed with aq. sat. NaHCO_3_ (2 × 20 ml) and aq. sat.NH_4_Cl (1 × 20 ml) and dried over Na_2_SO_4_. The solvent was removed *in vacuo*. The residue was purified by column chromatography on silica gel (DCM:MeOH, 95:5) to afford **9** (211 mg, 46%) as a white powder.

^1^H NMR (300 MHz, DMSO-d_6_) *δ* = 1.36 (t, 3H, *J* = 7.0 Hz), 3.79 (q, 2H, *J* = 7.0 Hz), 7.35–7.38 (m, 4H), 7.89 (d, 2H, *J =* 8.2 Hz) ppm ^13^C NMR (75 MHz, DMSO-d_6_) *δ* = 13.3, 44.6, 113.0, 116.4, 128.6, 139.6, 143.5 ppm HRMS (ESI) [M - H]^-^: *m*/*z* calcd for (C_9_H_10_N_3_O_2_S) 224.0494. Found 224.0499.

#### 4-(N-Butylcyanamido)benzenesulfonamide (10)


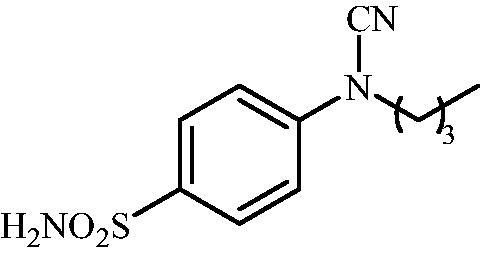


To a solution of methyl (4-sulfamoylphenyl)carbamimidothioate (**5**) (500 mg, 2.04 mmol) in DMF (8 ml) under stirring K_2_CO_3_ (564 mg, 4.08 mmol) was added in one portion The mixture was stirred at 100 °C for 1.5 h. After cooling to room temperature, *n*BuI (0.232 ml, 2.04 mmol) was added and the mixture was stirred at 100 °C for 1 h. The reaction mixture was cooled to room temperature and extracted with EtOAc (3 × 20 ml). The combined organic phases were washed with aq. sat. NaHCO_3_ (2 × 20 ml) and aq. sat.NH_4_Cl (1 × 20 ml) and dried over Na_2_SO_4_. The solvent was removed *in vacuo*. The residue was purified by column chromatography on silica gel (DCM:MeOH, 95:5) to afford **10** (279 mg, 54%) as a white powder.

^1^H NMR (300 MHz, DMSO-d_6_) *δ* = 0.96 (t, 3H, *J =* 7.1 Hz), 1.37–1.49 (m, 2H), 1.68–1.77 (m, 2H), 3.77 (t, 2H, *J =* 7.1 Hz), 7.37–7.39 (m, 4H), 7.88 (d, 2H, *J =* 8.7 Hz) ppm ^13^C NMR (75 MHz, DMSO-d_6_) *δ* = 14.5, 20.0, 29.8, 49.3, 113.4, 116.4, 128.6, 139.6, 143.6 ppm HRMS (ESI) [M]^+^: *m*/*z* calcd for (C_11_H_15_N_3_O_2_S) 253.0885. Found 253.0888.

#### 4-(N-Hexylcyanamido)benzenesulfonamide (11)


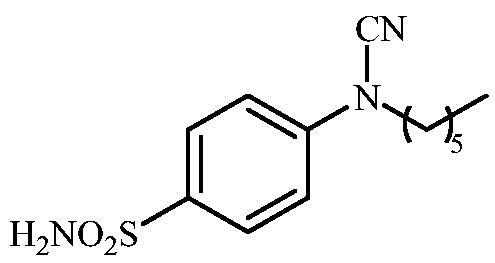


To a solution of methyl (4-sulfamoylphenyl)carbamimidothioate (**5**) (500 mg, 2.04 mmol) in DMF (8 ml) under stirring K_2_CO_3_ (564 mg, 4.08 mmol) was added in one portion. The mixture was stirred at 100 °C for 1.5 h. After cooling to room temperature, 1-bromohexane (0.232 ml, 2.04 mmol) and KI (372 mg, 2.24 mmol) were added and the mixture was stirred at 100 °C for 1.5 h. The reaction mixture was cooled to room temperature and extracted with EtOAc (3 × 20 ml). The combined organic phases were washed with aq. sat. NaHCO_3_ (2 × 20 ml) and aq. sat.NH_4_Cl (1 × 20 ml) and dried over Na_2_SO_4_. The solvent was removed *in vacuo*. The residue was purified by column chromatography on silica gel (DCM:MeOH, 95:5) to afford **11** (379 mg, 66%) as a white powder.

^1^H NMR (300 MHz, DMSO-d_6_) *δ* = 0.89 (t, 3H, *J* = 6.5 Hz), 1.33–1.44 (m, 6H), 1.69–1.78 (m, 2H), 3.77 (t, 2H, *J* = 6.5 Hz), 7.37–7.39 (m, 4H), 7.86 (d, 2H, *J* = 7.9 Hz) ppm ^13^C NMR (75 MHz, DMSO-d_6_) *δ* = 14.8, 22.9, 26.3, 27.7, 31.7, 49.5, 113.4, 116.4, 128.6, 139.8, 143.5 ppm HRMS (ESI) [M - H]^-^: *m*/*z* calcd for (C_13_H_18_N_3_O_2_S) 280.1120. Found 280.1124.

#### 4-(N-Heptylcyanamido)benzenesulfonamide (12)


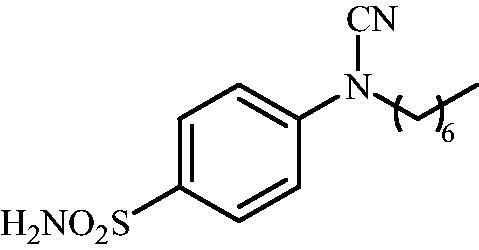


To a solution of methyl (4-sulfamoylphenyl)carbamimidothioate (**5**) (500 mg, 2.04 mmol) in DMF (8 ml) under stirring K_2_CO_3_ (564 mg, 4.08 mmol) was added in one portion The mixture was stirred at 100 °C for 1.5 h. After cooling to room temperature, 1-iodoheptane (0.335 ml, 2.04 mmol) was added and the mixture was stirred at 100 °C for 1.5 h. The reaction mixture was cooled to room temperature and extracted with EtOAc (3 × 20 ml). The combined organic phases were washed with aq. sat. NaHCO_3_ (2 × 20 ml) and aq. sat.NH_4_Cl (1 × 20 ml) and dried over Na_2_SO_4_. The solvent was removed *in vacuo*. The semisolid residue was dissolved in EtOAc (5 ml) and precipitated by addition of hexane (50 ml). The precipitate formed was filtered, washed with hexane and air dried to afford **12** (290 mg, 48%) as a white powder.

^1^H NMR (300 MHz, DMSO-d_6_) *δ* = 0.89 (t, 3H, *J* = 6.5 Hz), 1.29 (br s. 5H), 1.37 (br s, 3H), 1.69–1.78 (m, 2H), 3.76 (t, 2H, *J* = 6.9 Hz), 7.36–7.39 (m, 4H), 7.88 (d, 2H, *J =* 8.6 Hz) ppm ^13^C NMR (75 MHz, DMSO-d_6_) *δ* = 14.9, 22.9, 26.6, 27.7, 29.2, 32.1, 49.5, 113.4, 116.4, 128.6, 139.6, 143.5 ppm HRMS (ESI) [M - H]^-^: *m*/*z* calcd for (C_14_H_20_N_3_O_2_S) 294.1276. Found 294.1285.

#### 4-(N-Octadecylcyanamido)benzenesulfonamide (13)


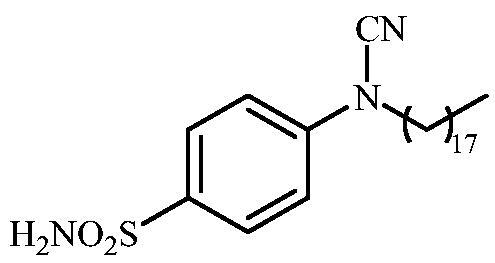


To a solution of methyl (4-sulfamoylphenyl)carbamimidothioate (**5**) (500 mg, 2.04 mmol) in DMF (8 ml) under stirring K_2_CO_3_ (564 mg, 4.08 mmol) was added in one portion The mixture was stirred at 100 °C for 1.5 h. After cooling to room temperature, 1-bromooctadecane (0.335 ml, 2.04 mmol) and KI (680 mg, 2.24 mmol) were added and the mixture was stirred at 100 °C for 1.5 h. The reaction mixture was cooled to room temperature and extracted with EtOAc (3 × 20 ml). The combined organic phases were washed with aq. sat. NaHCO_3_ (2 × 20 ml) and aq. sat.NH_4_Cl (1 × 20 ml) and dried over Na_2_SO_4_. The solvent was removed *in vacuo*. Residual yellowish solids were washed with cold EtOAc (15 ml) to afford **13** (165 mg, 18%) as a white powder.

^1^H NMR (300 MHz, DMSO-d_6_) *δ* = 0.88 (t, 3H, *J* = 6.7 Hz), 1.26 (br s, 30H), 1.68–1.77 (m, 2H), 3.75 (t, 2H, *J* = 6.7 Hz), 7.35–7.38 (m, 4H), 7.88 (d, 2H, *J =* 8.6 Hz) ppm ^13^C NMR (75 MHz, DMSO-d_6_) *δ* = 14.9, 23.1, 26.7, 27.8, 29.5, 29.7, 30.0 (br), 32.3, 49.5, 113.4, 116.4, 128.6, 139.6, 143.5 ppm HRMS (ESI) [M - H]^-^: *m*/*z* calcd for (C_25_H_42_N_3_O_2_S) 448.2998. Found 448.2994.

#### 4-(N-Cyclohexylcyanamido)benzenesulfonamide (14)


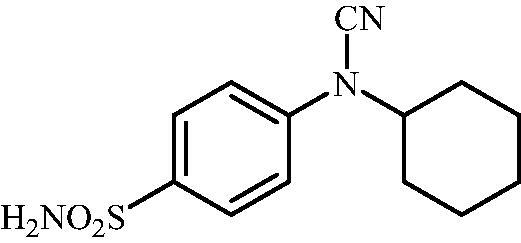


To a solution of methyl (4-sulfamoylphenyl)carbamimidothioate (**5**) (500 mg, 2.04 mmol) in DMF (8 ml) under stirring K_2_CO_3_ (564 mg, 4.08 mmol) was added in one portion The mixture was stirred at 100 °C for 1.5 h. After cooling to room temperature, bromocyclohexane (0.252 ml, 2.04 mmol) and KI (372 mg, 2.24 mmol) were added and the mixture was stirred at 100 °C for 1.5 h. The reaction mixture was cooled to room temperature and extracted with EtOAc (3 × 20 ml). The combined organic phases were washed with aq. sat. NaHCO_3_ (2 × 20 ml) and aq. sat.NH_4_Cl (1 × 20 ml) and dried over Na_2_SO_4_. The solvent was removed *in vacuo*. The residue was purified by column chromatography on silica gel (DCM:MeOH, 95:5) to afford **14** (85 mg, 15%) as a white powder.

^1^H NMR (300 MHz, DMSO-d_6_) *δ* = 1.19–1.27 (m, 1H), 1.41–1.55 (m, 4H), 1.68 (d, 1H, *J* = 12.1 Hz), 1.83 (s, 2H), 2.02 (s, 2H), 3.99 (s, 1H), 7.38 (s, 2H), 7.43 (d, 2H, *J* = 8.6 Hz), 7.88 (d, 2H, *J =* 8.6 Hz) ppm ^13^C NMR (75 MHz, DMSO-d_6_) *δ* = 25.4, 25.6, 31.5, 56.8, 111.9, 117.1, 128.6, 139.8, 143.3 ppm HRMS (ESI) [M - H]^-^: *m*/*z* calcd for (C_13_H_16_N_3_O_2_S) 278.0963. Found 278.0969.

#### 4-(N-Allylcyanamido)benzenesulfonamide (15)


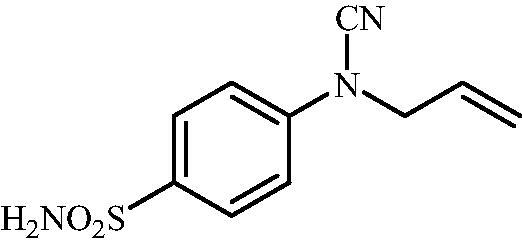


To a solution of methyl (4-sulfamoylphenyl)carbamimidothioate (**5**) (500 mg, 2.04 mmol) in DMF (8 ml) under stirring K_2_CO_3_ (564 mg, 4.08 mmol) was added in one portion The mixture was stirred at 100 °C for 1.5 h. After cooling to room temperature, allyl bromide (0.176 ml, 2.04 mmol) was added and the mixture was stirred at 100 °C for 1.5 h. The reaction mixture was cooled to room temperature and extracted with EtOAc (3 × 20 ml). The combined organic phases were washed with aq. sat. NaHCO_3_ (2 × 20 ml) and aq. sat.NH_4_Cl (1 × 20 ml) and dried over Na_2_SO_4_. The solvent was removed *in vacuo*. The semisolid residue was dissolved in EtOAc (5 ml) and precipitated by addition of hexane (50 ml). The precipitate formed was filtered, washed with hexane and air dried to afford **15** (290 mg, 60%) as a white powder.

^1^H NMR (300 MHz, DMSO-d_6_) *δ* = 4.46 (d, 2H, *J* = 4.9 Hz), 5.36 (s, 1H), 5.41 (d, 1H, *J* = 7.8 Hz), 5.94–6.06 (m, 1H), 7.36 (d, 2H, *J* = 8.6 Hz), 7.38 (s, 2H), 7.88 (d, 2H, *J =* 8.6 Hz) ppm ^13^C NMR (75 MHz, DMSO-d_6_) *δ* = 52.0, 113.4, 116.5, 120.5, 128.5, 131.6, 139.7, 143.3 ppm HRMS (ESI) [M - H]^-^: *m*/*z* calcd for (C_10_H_10_N_3_O_2_S) 236.0494. Found 236.0489.

#### 4-(N-Benzylcyanamido)benzenesulfonamide (16)


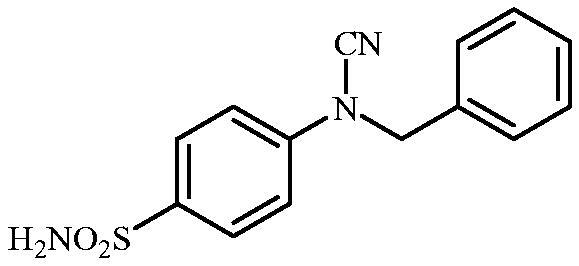


To a solution of methyl (4-sulfamoylphenyl)carbamimidothioate (**5**) (500 mg, 2.04 mmol) in DMF (8 ml) under stirring K_2_CO_3_ (564 mg, 4.08 mmol) was added in one portion The mixture was stirred at 100 °C for 1.5 h. After cooling to room temperature, benzyl bromide (0.236 ml, 2.04 mmol) was added and the mixture was stirred at 100 °C for 1.5 h. The reaction mixture was cooled to room temperature and water was added until precipitate was formed. The precipitate was filtered, washed with water (10 ml) and Et_2_O (10 ml) and air dried to afford **16** (282 mg, 48%) as a white powder.

^1^H NMR (300 MHz, DMSO-d_6_) *δ* = 5.06 (s, 2H), 7.35–7.44 (m, 9H), 7.86 (d, 2H, *J =* 8.4 Hz) ppm ^13^C NMR (75 MHz, DMSO-d_6_) *δ* = 52.5, 113.2, 116.2, 128.1, 128.3, 128.8, 129.4, 135.0, 139.3, 142.8 ppm HRMS (ESI) [M - H]^-^: *m*/*z* calcd for (C_14_H_12_N_3_O_2_S) 286.0650. Found 286.0649.

#### 4-(N-(4-Bromobenzyl)cyanamido)benzenesulfonamide (17)


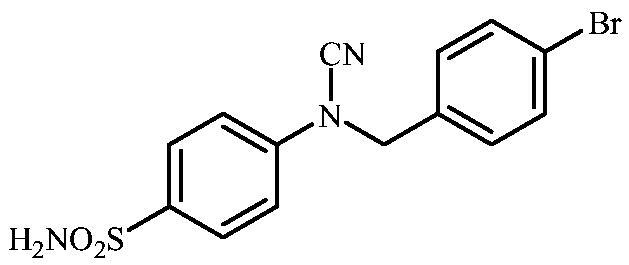


To a solution of methyl (4-sulfamoylphenyl)carbamimidothioate (**5**) (500 mg, 2.04 mmol) in DMF (8 ml) under stirring K_2_CO_3_ (564 mg, 4.08 mmol) was added in one portion The mixture was stirred at 100 °C for 1.5 h. After cooling to room temperature, 4-bromobenzyl bromide (659 mg, 2.04 mmol) was added and the mixture was stirred at 100 °C for 1.5 h. The reaction mixture was cooled to room temperature and water was added until precipitate was formed. The precipitate was filtered, washed with water (10 ml) and Et_2_O (10 ml) and air dried to afford **17** (501 mg, 67%) as a white powder.

^1^H NMR (300 MHz, DMSO-d_6_) *δ* = 5.06 (s, 2H), 7.36–7.42 (m, 6H), 7.65 (d, 2H, *J =* 8.0 Hz), 7.87 (d, 2H, *J =* 8.0 Hz) ppm ^13^C NMR (75 MHz, DMSO-d_6_) *δ* = 52.3, 113.5, 116.7, 122.6, 128.6, 131.1, 132.8, 134.9, 139.9, 143.2 ppm HRMS (ESI) [M - H]^-^: *m*/*z* calcd for (C_14_H_11_N_3_O_2_SBr) 363.9755. Found 363.9758.

### CA inhibitory assay

An applied photophysics stopped-flow instrument has been used for assaying the CA catalysed CO_2_ hydration activity^22^. Phenol red (at a concentration of 0.2 mM) was used as indicator, working at the absorbance maximum of 557 nm, with 20 mM Hepes (pH 7.5) as buffer for α-CAs or 20 mM TRIS (pH 8.4) as buffer for β-CAs, and 20 mM Na_2_SO_4_ (for maintaining constant the ionic strength), following the initial rates of the CA-catalysed CO_2_ hydration reaction for a period of 10 − 100 s. The CO_2_ concentrations ranged from 1.7 to 17 mM for the determination of the kinetic parameters and inhibition constants. For each inhibitor, at least six traces of the initial 5 − 10% of the reaction have been used for determining the initial velocity. The uncatalysed rates were determined in the same manner and subtracted from the total observed rates. Stock solutions of inhibitor (0.1 mM) were prepared in distilled – deionised water, and dilutions up to 0.01 nM were done thereafter with the assay buffer. Inhibitor and enzyme solutions were preincubated together for 6 h at room temperature prior to assay in order to allow for the formation of the E – I complex. The inhibition constants were obtained by nonlinear least-squares methods using PRISM 3 and the Cheng – Prusoff equation, as reported earlier^23–25^ and represent the mean from at least three different determinations. All CA isoforms were recombinant ones obtained in-house as reported earlier^26–28^ and their concentrations in the assay system ranged between 8–15 nM.

## Results and discussion

### Chemistry

The drug design of the new CAIs reported here considered the benzenesulphonamide scaffold, which has been widely employed earlier for derivatization reactions, mainly by using the tail approach, as it allows a facile chemistry and generally high yields of new products^2–4^. The general synthetic route of the target compounds is shown in Scheme 3. In order to bypass the need for toxic cyanogen bromide, a multi-step procedure was designed which involves the initial formation of methyl (4-sulfamoylphenyl) carbamimidothioate **5** via a two-step procedure through thioureation of sulphanilamide **SA** (4-amino-benzenesulfonamide) using KSCN and subsequent *S*-methylation of the resulted thiourea **4** with MeI. The elimination of methyl sulphide from carbamimidothioate **5** by treatment with K_2_CO_3_ afforded potassium cyano(4-sulphamoylphenyl)amide **6** in excellent yield which by treatment with acid was converted into the corresponding secondary cyanamide **7**. The target 4-(*N*-alkyl/benzyl-cyanamido)benzenesulfonamide derivatives **8–17** were synthesised by reaction of *in situ* generated potassium cyano(4-sulfamoylphenyl)amide **6** with selected alkyl/benzyl halides (iodides or bromides) under catalyst- and additive-free conditions in DMF at elevated temperatures (Scheme 3).

### Carbonic anhydrase inhibition

The cyanamido-benzenesulfonamides **6–17** reported here have been tested as inhibitors of three human (h) CA isoforms, the cytosolic hCA I, II, VII and XIII, as well as the bacterial β-CAs from *Mammaliicoccus (Staphylococcus) sciuri*^26^ and *Salmonella enterica* (serovar *Typhimurium*), StCA1 and StCA2^27^. It should be mentioned that the first bacterial enzyme, SscCA, was originally reported by us as *Staphylococcus aureus* β-CA, SauCA based on a genomic sequence annotated in the data bases in 2017^26^. A recent reanalysis of that sequence revealed that the original annotation was erroneous, and that the sequence encodes a β-class CA from another *Staphylococcaceae* family member, i.e. *Staphylococcus sciuri*, which is a Gram-positive, oxidase-positive, coagulase-negative member of these infectious bacteria known to provoke disease in humans and animals (it was originally isolated from the squirrel)^29^. In 2020, Madhaiyan et al. renamed the species as belonging to a new genus, as *Mammaliicoccus sciuri*^30^. In fact, the taxonomy of the *Staphylococcaceae* family is rather complex, and as mentioned earlier, many genome annotations were inexact or were overlapping between various genetically similar species^30^. Thus, we report here that the enzyme previously known as SauCA is in fact MscCA^31^.

The following should be noted regarding data of Table 1, where the inhibition data of these enzymes are presented:

**Table 1.** Inhibition data of human CA isoforms CA I, II, VII, and XIII and bacterial β-CA isoforms SscCA, from *Mammaliicoccus (Staphylococcus) sciuri*, and StCA1 and StCA2, from *Salmonella enterica* (serovar *Typhimurium*), with compounds **6–17** using acetazolamide (**AAZ**) as standard drug. 
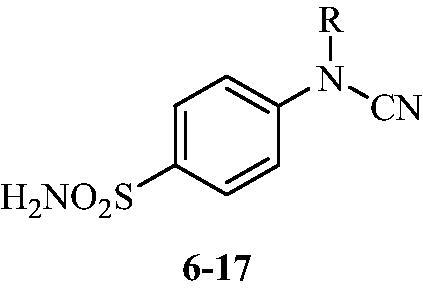

Cmpd	R	K_I_ (nM)^a^						
		hCA I (α-CA)	hCA II (α-CA)	hCA VII (α-CA)	hCA XIII (α-CA)	MscCA (β-CA)	StCA1 (β-CA)	StCA2 (β-CA)
**6**	K^+^	867	130	30.6	62.6	860	87.7	92.4
**7**	–H	889	148	22.7	50.5	870	91.1	94.7
**8**	–CH_3_	557	29.2	13.8	117	1585	52.7	200
**9**	–CH_2_CH_3_	88.6	45.3	17.9	476	3151	61.5	367
**10**	–(CH_2_)_3_CH_3_	16.4	32.0	1.3	70.7	4476	62.7	426
**11**	–(CH_2_)_5_CH_3_	9.3	5.3	1.5	83.0	6885	54.6	718
**12**	–(CH_2_)_6_CH_3_	38.2	7.1	1.7	31.5	7188	58.8	676
**13**	–(CH_2_)_17_CH_3_	17.3	9.5	2.2	155	9173	66.1	791
**14**	–Cy	50.6	82.3	1.9	83.8	5359	69.5	579
**15**	–CH_2_CH = CH_2_	74.7	54.1	2.3	97.7	5096	51.2	645
**16**	–CH_2_C_6_H_5_	67.3	61.4	2.0	67.8	9361	50.7	692
**17**	–CH_2_(4–Br–C_6_H_4_)	389	93.3	2.4	53.1	9592	73.1	750
**AAZ**	–	250	12.5	2.5	16.0	625	59.0	84

^a^Mean from 3 different assays, by a stopped flow technique (errors were in the range of ± 5–10% of the reported values).

- hCA I was effectively inhibited by cyanamido-benzenesulphonamides **6–17** reported here with K_I_s ranging between 9.3 and 889 nM (acetazolamide, the standard CAI is a medium potency inhibitor with a K_I_ of 250 nM). The most effective hCA I inhibitors in the series were **10–14**, K_I_s of 9.3–50.6 nM, all of which incorporate rather long aliphatic R moieties at the cyanamide functionality. The optimal substitution seems to be an *n*-hexyl group, in **11**, which is the most effective hCA I inhibitor reported here. Smaller aliphatic R moieties, unsaturated or aromatic ones lead to a decrease of the hCA I inhibitory potency.
- hCA II is also effectively inhibited by these sulphonamides, with K_I_s ranging between 5.3 and 148 nM (Table 1). Derivatives **11–13** were the most effective inhibitors, with K_I_s of 5.3–9.5 nM, even better than **AAZ** (K_I_ of 12 nM). Again the presence of long aliphatic chains (C6, C7 and C18) induced these effective inhibitory effects, whereas shorter, unsaturated or aromatic/benzylic R groups reduce (in some cases only slightly) the potency.
- hCA VII, the mainly brain expressed cytosolic isoform was the most susceptible to inhibition with the newly prepared sulphonamides, which showed K_I_s ranging between 1.3 and 30.6 nM (Table 1). Thus, all substitution patterns present in these compounds lead to highly effective CAIs, with the optimal substitutions being those present in **10–17**. Thus, for this isoform, apart the long aliphatic groups, the unsaturated, benzylic and substituted benzyl moieties afforded highly effective inhibitors.
- MscCA was on the other hand poorly inhibited by the cyanamido-benzenesulphonamides **6–17** reported here, with K_I_s in the micromolar range, more precisely 860–9592 nM (Table 1). The best inhibitors were the unsubstituted cyanamides **6** and **7**, which like acetazolamide, are medium potency bacterial CA inhibitors.
- StCA1 was the best inhibited bacterial CA among the three such enzymes investigated here with cyanamido-benzenesulphonamides **6–17.** Indeed, these compounds showed K_I_s ranging between 50.7 and 91.1 nM. Thus, all of them show a quite flat structure-activity relationship, acting as effective (but not highly potent) CAIs.
- The second *S. enterica* (serovar *Typhimurium*) CA isoform StCA2 was on the other hand less sensitive to inhibition with these new sulphonamides compared to StCA1, and the K_I_s were in the range of 92.4–791 nM. In this case, the best inhibitors were the ones with small R groups (H or potassium), compounds **6** and **7**, whereas the increase of the R moiety led to a decrease of the inhibitory power (Table 1).

## Conclusions

A one-pot two-step protocol was developed for the synthesis of novel 4-cyanamidobenzenesulfonamide derivatives from easily accessible methyl (4-sulfamoylphenyl)carbamimidothioate under catalyst- and additive-free conditions. The small series of prepared compounds was investigated for the inhibition of human and bacterial CAs belonging to the α- and β-CA classes. Several highly effective hCA I, II, and VII inhiibtors were detected, whereas hCA XIII was less inhibited by most such compounds. The structure-activity relationship for the inhibition of the human isoforms is rather straightforward, with long aliphatic chains (C6, C7 and C18) at the cyanamide functionality inducing the most effective inhibitory effects. Among the three bacterial CAs investigated here for their inhibition with cyanamidobenzenesulfonamides, StCA1 was the most sensitive to these inhibitors, followed by StCA2, whereas MscCA was inhibited only in the micromolar range. Although these compounds do not show selectivity for the inhibition of bacterial versus human CAs, they may be considered as interesting starting points for the development of novel pharmacological agents belonging to this class of enzyme inhibitors.
